# Assessment of inpatient multimodal cardiac imaging appropriateness at large academic medical centers

**DOI:** 10.1186/s12947-015-0037-0

**Published:** 2015-11-14

**Authors:** Andrew Remfry, Howard Abrams, David M. Dudzinski, Rory B. Weiner, R. Sacha Bhatia

**Affiliations:** University of Toronto Medical School, Medical Sciences Building, 1 King’s College Circle, Toronto, M5S 1A8 Canada; Peter Munk Cardiac Centre of the University Health Network, Toronto General Hospital, 200 Elizabeth St, Toronto, ON M5G 2C4 Canada; Massachusetts General Hospital, 55 Fruit Sreet, Boston, MA 02114 USA; Women’s College Hospital Institute for Health Systems Solutions and Virtual Care, 76 Grenville Street, Toronto, ON M5S 1B2 Canada; Adjunct Scientist, Institute for Clinical Evaluative Sciences, Division of Cardiology, University Health Network and Women’s College Hospital, University of Toronto, 76 Grenville Street, 6th Floor, Toronto, ON M5S 1B2 Canada

**Keywords:** Appropriateness use criteria, transthoracic echocardiogram, Cardiac catheterization, transesophageal echocardiogram, Single-photon emission tomography myocardial perfusion imaging

## Abstract

**Background:**

Responding to concerns regarding the growth of cardiac testing, the American College of Cardiology Foundation (ACCF) published Appropriate Use Criteria (AUC) for various cardiac imaging modalities. Single modality cardiac imaging appropriateness has been reported but there have been no studies assessing the appropriateness of multiple imaging modalities in an inpatient environment.

**Methods:**

A retrospective study of the appropriateness of cardiac tests ordered by the inpatient General Internal Medicine (GIM) and Cardiology services at three Canadian academic hospitals was conducted over two one-month periods. Cardiac tests characterized were transthoracic echocardiography (TTE), transesophageal echocardiography (TEE), single-photon emission tomography myocardial perfusion imaging (SPECT), and diagnostic cardiac catheterization.

**Results:**

Overall, 553 tests were assessed, of which 99.8 % were classifiable by AUC. 91 % of all studies were categorized as appropriate, 4 % may be appropriate and 5 % were rarely appropriate. There were high rates of appropriate use of all modalities by GIM and Cardiology throughout. Significantly more appropriate diagnostic catheterizations were ordered by Cardiology than GIM (93 % vs. 82 %, *p* = <0.01). Cardiology ordered more appropriate studies overall (94 % vs. 88 %, *p* = 0.03) but there was no difference in the rate of rarely appropriate studies (3 % vs. 6 %, *p* = 0.23).

**Conclusion:**

The ACCF AUC captured the vast majority of clinical scenarios for multiple cardiac imaging modalities in this multi-centered study on Cardiology and GIM inpatients in the acute care setting. The rate of appropriate ordering was high across all imaging modalities. We recommend further work towards improving appropriate utilization of cardiac imaging resources focus on the out-patient setting.

## Background

Advances in cardiac imaging have led to vast improvements in our ability to diagnose heart disease. These advances have led to an increase in cardiac imaging utilization and healthcare spending in this area as a result [1, 2]. In response to concerns regarding increased utilization, the American College of Cardiology Foundation (ACCF), in conjunction with other specialty societies, published appropriate use criteria (AUC) for various imaging modalities, including transthoracic (TTE) and transesophageal echocardiography (TEE), radionuclide imaging and diagnostic cardiac catheterization [3–5]. These initially classified studies as ‘appropriate’, ‘may be appropriate’ or ‘rarely appropriate’, but subsequently updated the terms to ‘appropriate’, ‘may be appropriate’ and ‘rarely appropriate’ [6]. Previous retrospective studies using the AUC to classify imaging have identified rates of up to 22 % for TTE in the category of rarely appropriate, from a variety of clinical settings [7–10]. Appropriateness studies of single-photon emission tomography myocardial perfusion imaging (SPECT), and cardiac catheterization have shown similar rates of rarely appropriate use [11–13].

While most studies have focused on assessing appropriate use rates for individual cardiac imaging modalities, no prior studies have examined the rates of appropriateness of multiple cardiac imaging modalities simultaneously. To better understand appropriateness of multi-modal cardiac imaging in the acute care setting, we performed a retrospective review of in-patients undergoing a range of cardiac investigations at multiple large academic medical centers in Toronto, Canada, using the AUC for the most common cardiac imaging modalities. We hypothesized that the combined rate of ‘rarely appropriate’ use for several combined imaging modalities would be comparable to prior studies of single imaging modalities in other countries.

## Methods

### Study design

We conducted a retrospective analysis of 553 consecutive cardiac investigations requested by physicians attending on the General Internal Medicine (GIM) and Cardiology in-patient services across three academic teaching hospitals in Toronto. The study was conducted in two separate 1-month blocks (26th August – 22nd September 2013 and 10th February – 8th March 2014). Approval was received from Research Ethics Boards at each hospital.

### Study hospitals and participants

Three sites were included in this study: Toronto General Hospital (TGH), Toronto Western Hospital (TWH) and Mount Sinai Hospital (MSH). At each site, we studied ordering of cardiac imaging on both the GIM and general Cardiology inpatient services. Each hospital has on-site TTE, SPECT, and TEE services. Two hospitals (TGH, TWH) had on-site cardiac catheterization laboratories. The third (MSH) did not have an on-site laboratory, but was physically connected to another lab (TGH), and access to that lab was readily available for MSH patients. Although Cardiac Computed Tomography (CT) and cardiac Magnetic Resonance Imaging (MRI) were available at all sites, we did not include those modalities because the inpatient ordering volumes of those tests were low.

For GIM services, there are five inpatient teams at each site, each staffed by residents, medical students and one attending physician. The general Cardiology services are composed of medical residents, one cardiology fellow and one attending at two sites (MSH and TWH), and a team of nurse practitioners, cardiology fellows and one cardiology attending at the third site (TGH). Staff cardiologists attend for one week at a time at each site, and GIM staff attend for 2 to 4 weeks at a time. On both the Cardiology and GIM services, investigations can be requested by any member of the medical team except for medical students, who require orders to be co-signed by a resident or attending staff.

We identified cardiac investigations conducted on patients admitted to the three sites over the study period. Representatives from the on-service GIM and Cardiology teams at each site were contacted on each weekday of the study period to identify patients who had undergone TTE, TEE, nuclear perfusion study or cardiac catheterization in the previous 24 h. Patients who had a study performed over the weekend were identified on a Monday morning. All investigations ordered by the attending team on in-patients during the study period were included, except for patients aged < 18 years old, or where there was insufficient information available from the electronic medical record (EMR) or patient chart to ascertain the indication for the study.

### Data collection

Patient demographics and clinical reasons for requesting the investigation were recorded from a combination of the patient’s written chart (including admission note, consults and daily entries) and the EMR. The EMR is a comprehensive medical record that includes medical notes, prior medical imaging reports and laboratory tests. There were no decisional support tools for the ordering of cardiac investigations at any site.

Data was collected and appropriateness category assigned (appropriate, may be appropriate, or rarely appropriate) by the principal investigator (A.R.), a trainee in Internal Medicine with no connection to the cardiac imaging service at any site. A secondary reviewer (R.S.B) is a level three Echocardiographer with a special interest in the Appropriate Use Criteria. We used the most recent Appropriate Use Criteria for each of the individual modalities. Studies that did not have an associated AUC clinical scenario were considered unclassified. Cases where there was uncertainty regarding the classification of a study were reviewed with the second reviewer and consensus was reached, though this occurred fewer than 5 times over the study period. RSB conducted a blinded classification of a random sample of 262 cardiac imaging studies and found concordance of 96 % for TTE, 93 % for TEE, 92 % for SPECT, and 96 % for cardiac catheterization.

### Statistical analysis

Categorical variables for AUC rating and patient demographics were compared between sites and between services (GIM and Cardiology) using *χ*^*2*^ or Fishers exact test. Continuous variables are reported as the mean plus standard deviation and were compared across practice sites using analysis of variance. Statistical significance was indicated by two-tailed *P* < 0.05.

## Results

### Study population

In total, 553 cardiac investigations were reviewed across the three different practice sites; 142 from site 1, 248 from site 2 and 163 from site 3. When separated into specialty there were 277 investigations requested by the GIM service and 276 by the cardiology service. No investigations were excluded from this study.

### Patient demographics

The patient demographics for all three study sites are presented in Table 1. Overall, the patients’ burden of co-morbidities was typical of patients seen on both a general Cardiology and GIM service. Patients at site 1 were generally older but with lower rates of hypertension, hyperlipidaemia, valvular heart disease and angina than the other two sites, with fewer cardiac investigations or interventions. As might be expected, Cardiology inpatients had a generally higher prevalence of cardiac risk factors as well as a higher rate of prior cardiac investigations and interventions.

**Table 1:** Patient characteristics

Characteristics	Site 1	Site 2	Site 3	*P* value	GIM	Cardiology	*P* value
Patients (n)	142	248	163		277	276	
Age (SD)	*69.5 (18.2)	*65.3 (15.2)	68.4 (16.3)	*< 0.03	68.6 (16.6)	66.1 (16.2)	0.08
Male (%)	49	56	54	0.44	54	56	0.37
HTN (%)	60	67	77	0.01	68	68	0.92
Smoker (%)	21	27	33	0.06	32.5	22.5	0.01
Hyperlipidaemia (%)	39	54	52	0.02	39.4	59.8	<0.01
Chronic kidney disease (%)	11	12	18	0.12	13.7	13.8	0.92
Diabetes (%)	27	30	37	0.14	28.2	34.1	0.16
Angina (%)	4	18	15	0.01	7.3	19.6	<0.01
Prior ACS (%)	14	23	23	0.09	15.9	25	0.01
Prior PCI (%)	8	25	16	<0.01	9.7	26.8	<0.01
Prior CABG (%)	6	11	10	0.27	6.9	12.7	0.03
Congestive Heart Failure (%)	22	14	23	0.04	19.5	18.1	0.76
Valvular heart disease (%)	8	18	10	0.01	9.7	16.3	0.0303
Prior TTE (%)	48	57	56	0.17	45	64	<0.01
Prior TEE (%)	1	8	3	0.01	2	7	0.01
Prior SPECT (%)	6	23	30	<0.01	16	26	<0.001
Prior angiogram (%)	20	46	33	<0.01	18	53	<0.01

Patient demographics and clinical information from each study population. *P* values for differences between sites and between GIM and Cardiology are shown. *HTN* Hypertension, *PCI* Percutaneous Coronary Intervention, *ACS* acute coronary syndrome, *CABG* coronary artery by-pass graft, *TTE* transthoracic echocardiography, *TEE* transesophageal echocardiography, *SPECT* single-photon emission tomography myocardial perfusion imaging. *p value relates to the difference between the average age of patient from site 1 and site 2

### Appropriateness classification

#### Transthoracic echocardiography

A total of 365 TTEs were ordered over the study period; 113 from site 1, 154 from site 2 and 98 from site 3. All were classifiable using the 2011 AUC for TTE. When all sites were combined, 210 TTEs were requested by GIM and 155 TTEs were requested by Cardiology. The combined results across all sites and specialties were 90 % appropriate, 3 % may be appropriate and 7 % rarely appropriate (Table 2). The overall rate of rarely appropriate TTE ordering was not significantly different between the three sites. Rarely appropriate TTE ordering was similar between GIM and Cardiology (8 and 6 %, *p* = 0.64). The most common appropriate, may be appropriate and rarely appropriate indications for TTE request are listed in Table 3.

**Table 2:** Appropriateness per site and specialty

Modality	Category	Site 1	Site 2	Site 3	*P* value	GIM	Cardiology	*P* value
TTE	Appropriate % (n)	87 (98)	92 (142)	91 (89)	0.32	88 (185)	93 (144)	0.18
	May be appropriate % (n)	4 (5)	1 (2)	4 (4)	0.26	4 (9)	1 (2)	0.18
	Rarely appropriate % (n)	9 (10)	6 (10)	5 (5)	0.55	8 (16)	6 (9)	0.64
TEE	Appropriate % (n)	100 (12)	100 (14)	100 (3)	N/A	100 (12)	100 (17)	N/A
	May be appropriate % (n)	0	0	0	N/A	0	0	N/A
	Rarely appropriate % (n)	0	0	0	N/A	0	0	N/A
SPECT	Appropriate % (n)	100 (1)	86 (12)	97 (30)	0.37	91 (30)	100 (13)	0.65
	May be appropriate % (n)	0	14 (2)	3 (1)	N/A	9 (3)	0	N/A
	Rarely appropriate % (n)	0	0	0	N/A	0	0	N/A
Diagnostic Catheterization	Appropriate % (n)	94 (15)	88 (57)	97 (30)	0.95	82 (18)	93 (84)	<0.01
	May be appropriate % (n)	6 (1)	12 (8)	3 (1)	0.31	18 (4)	7 (6)	0.2
	Rarely appropriate % (n)	0	0	0	N/A	0	0	N/A
Combined imaging	Appropriate % (n)	89 (126)	91 (225)	93 (152)	0.38	88 (245)	94 (258)	0.03
	May be appropriate % (n)	4 (6)	5 (12)	4 (6)	0.85	6 (16)	3 (8)	0.15
	Rarely appropriate % (n)	7 (10)	4 (10)	3 (5)	0.2	6 (16)	3 (9)	0.23

Table demonstrating the percentage of cardiac investigations in each appropriateness category (based on ACCF Appropriate Use Criteria) per site and between the General Internal Medicine (GIM) and Cardiology services. *P* values for differences in appropriate ordering between sites and between GIM and Cardiology are shown. *TTE* transthoracic echocardiography, *TEE* transesophageal echocardiography, *SPECT* single-photon emission tomography myocardial perfusion imaging

**Table 3:** Most common indications per imaging modality

Modality	Indication	n (%)
TTE (*n* = 365)		
Appropriate	Initial evaluation of known or suspected HF (systolic or diastolic) based on symptoms, signs, or abnormal test results	47 (13)
	Initial evaluation of ventricular function following ACS	38 (10)
	Initial evaluation of suspected infective endocarditis with positive blood cultures or a new murmur	29 (8)
May be appropriate	Re-evaluation of known HF (systolic or diastolic) with a change in clinical status or cardiac exam with a clear precipitating change in medication or diet	8 (2)
Rarely appropriate	Lightheadedness/presyncope when there are no other symptoms or signs of cardiovascular disease	4 (1)
	Infective endocarditis (native or prosthetic valves) with TTE: Transient bacteraemia with a pathogen not typically associated with infective endocarditis and/or a documented non endovascular source of infection	3 (1)
	Initial evaluation of ventricular function (e.g., screening) with no symptoms or signs of cardiovascular disease	3 (1)
TEE (*n* = 29)		
Appropriate	To diagnose infective endocarditis with a moderate or high pre-test probability (e.g., staph. bacteremia, fungemia, prosthetic heart valve, or intra-cardiac device)	8 (28)
	Atrial Fibrillation/Flutter: Evaluation to facilitate clinical decision making with regard to anticoagulation, cardioversion, and/or radiofrequency ablation Should add AUC 106?	8 (28)
SPECT (*n* = 46)		
Appropriate	Risk assessment with prior test results and/or known chronic stable CAD: New or worsening symptoms & abnormal coronary angiography OR abnormal prior stress imaging study	8 (17)
	Detection of CAD: acute chest pain; possible ACS with no ischemic changes or with LBBB or electronically ventricular paced rhythm, low-risk TIMI score & peak troponin borderline, equivocal or minimally elevated	6 (13)
May be appropriate	New or worsening symptoms; Normal coronary angiography OR normal prior stress imaging study	2 (4)
Diagnostic Catheterization (*n* = 112)		
Appropriate	Suspected or known ACS: UA/NSTEMI	64 (57)
	Valvular disease: Preoperative assessment before valvular surgery	9 (14)
May be appropriate	Suspected CAD: Prior non-invasive testing (no prior PCI, CABG, or angiogram showing >50 % angiographic stenosis); ECG stress testing with intermediate-risk findings (e.g., Duke treadmill score 4 to 10)	2 (2)
	Suspected CAD: Prior non-invasive testing (no prior PCI, CABG, or Angiogram Showing >50 % Angiographic Stenosis); Low-risk findings (e.g., 5 % ischemic myocardium on stress SPECT MPI or stress PET, no stress-induced wall motion abnormalities on stress echo or stress CMR) and symptomatic	2 (2)

Table listing the most commonly recorded indications in each appropriateness category for each imaging modality. *TTE* transthoracic echocardiography, *TEE* transesophageal echocardiography, *SPECT* single-photon emission tomography myocardial perfusion imaging

#### Transesophageal echocardiography

The number of TEEs ordered over the two collections periods was low across all three sites (29 in total) and all were for appropriate indications. The majority of TEEs were ordered at site 1 (*n* = 12) and site 2 (*n* = 14). All were classifiable using the AUC for echocardiography. The majority of TEEs were ordered for the diagnosis of infective endocarditis with a moderate to high pre-test probability (AUC #108), to facilitate decision making regarding anti-coagulation with respect to atrial fibrillation or flutter and cardioversion (AUC #112) and for the evaluation of valve structure and function prior to a procedure (AUC #106).

#### SPECT imaging

In total, 46 SPECT studies were ordered, with the largest number (31) from site 3. Overall more SPECT studies were performed by GIM than Cardiology (33 vs. 13). All were classifiable using the AUC for SPECT. There were no rarely appropriate studies and few studies that were classified as may be appropriate (2 at site 2 and 1 at site 3). The appropriateness rate between sites was similar (100 % at site 1 vs. 86 % at site 2 vs. 97 % at site 3, *p* = 0.37). There was also no difference in appropriateness of SPECT ordering between GIM and Cardiology (91 % vs. 100 %, *p* = 0.65). When all three sites were combined, the overall rate of appropriate ordering was 93 % and 7 % for studies that may be appropriate. The most common indications for requesting a SPECT study are listed in Table 3.

#### Diagnostic cardiac catheterization

A total of 113 cardiac catheterizations were ordered, of which 112 (99 %) were classifiable using the 2011 AUC. The bulk of these came from site 2 (65), followed by site 3 (31) and site 1 (16). There were no rarely appropriate studies and no significant difference between the high rates of appropriate ordering across all three sites (94 % vs. 88 % vs. 97 %, *p* = 0.95). Overall, the proportion of appropriate studies ordered was higher on the Cardiology service compared to the GIM service (93 % vs. 82 %, *p* < 0.01). There was no difference in the proportion of may be appropriate studies ordered at each at site (6 % vs. 12 % vs. 3 %, *p* = 0.31). The rate of may be appropriate studies was also not significantly different between GIM and Cardiology (18 % vs. 7 %, *p* = 0.2). When combined across all three sites, the rate of appropriate ordering was 91 %, while the remaining 9 % may have been appropriate. There were no rarely appropriate studies ordered. The most common appropriate and may be appropriate indications for requesting a cardiac catheterization are listed in Table 3.

#### Combined imaging appropriateness

The classification rate for all of the cardiac imaging modalities studies was high. Of the 553 cardiac imaging studies ordered, only one was not classifiable (99.8 % classified). Overall 91 % of all studies were appropriate, 4 % may be appropriate and 5 % were rarely appropriate. When all the imaging modalities are combined, there is no difference between appropriate rates of ordering between sites (89 %, vs. 91 % vs. 93 %, *p* = 0.38). The rates of may be appropriate and rarely appropriate ordering were also similar between sites. Between specialties, the proportion of appropriate studies ordered was higher for the Cardiology compared to the GIM service (94 % vs. 88 %, *p* = 0.03). The rates of may be appropriate and rarely appropriate ordering were not statistically different between GIM and Cardiology.

## Discussion

**Fig. 1:**
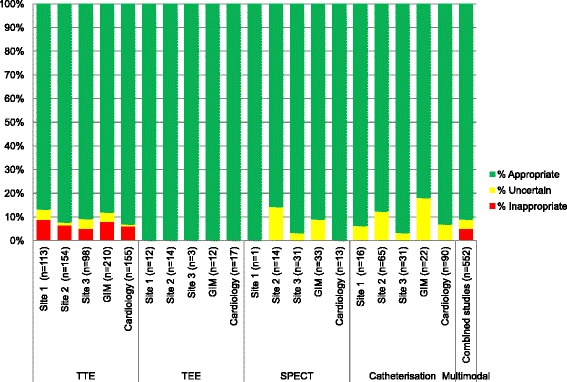
Chart demonstrating the proportion of appropriate, may be appropriate & rarely appropriate cardiac imaging investigations by site and specialty (based on ACCF Appropriate Use Criteria). GIM = General Internal Medicine, TTE = transthoracic echocardiography, TEE = transesophageal echocardiography, SPECT = single-photon emission tomography myocardial perfusion imaging

In this study we have presented data showing the application of AUC across a variety of imaging modalities in the in-patient population from three distinct academic teaching hospitals in a single payer healthcare system. To our knowledge, this is the first study to look specifically at the ordering practices of physicians in the acute care setting with respect to the range of cardiac imaging at their disposal. We have demonstrated that the recent versions of the AUC are capable of classifying the vast majority of clinical indications for cardiac imaging in the modern acute care setting. We also found that the combined rate of rarely appropriate ordering for cardiac investigations was low in all three study sites and between specialties, despite varying use of each imaging modality (Fig. 1).

A recognised limitation of many previous studies using AUC is that it can be difficult to determine from the written record the exact clinical indication for a particular test. In a large, single centre investigation into the appropriate use of transthoracic echocardiography, Bailey et al. [14], were able to achieve high levels of clinical reasoning determination (92 % of the cases) by undertaking a comprehensive review of admission history and physical examination, specialist consults, laboratory results and discharge summaries. In our work, we believe that by reviewing the daily chart entries in addition to the admission consult and electronic data, we were better able to appreciate the medical team’s reasoning and thus were confident in our ability to determine the clinical indication for testing in all the cases we reviewed. We were able to find a suitable clinical category for all but one the cases reviewed in the most up to date AUC for each imaging modality. This is similar to many other studies in the US and Europe which have found that the most recent AUC for echocardiography, SPECT and diagnostic catheterization are able to capture the vast majority of clinical situations and generally have high rates of classification [7–9, 12, 13, 15, 16].

‘The low rates of rarely appropriate use we observed likely represent the fact that the acute change in clinical status that brings most patients to hospital often justifies in-hospital cardiac imaging. This is reflected in the acuity of the most common indications for testing in each modality, which broadly included a new diagnosis of heart failure (TTE AUC #70), suspicion for infective endocarditis (TTE AUC #52 and TEE AUC #108), confirmed or suspected ACS with adjunctive work up (diagnostic catheterization AUC #3, TTE AUC #24 and SPECT AUC #6), new arrhythmia (TEE AUC #112) or worsening symptoms in patients with known coronary disease (SPECT AUC #30). This has been noted in previous studies, such as that by Matulevicius et al. [10], who noted higher rates of appropriate trans-thoracic echo in in-patients compared to out-patients. Studies looking at perfusion stress imaging have generally not separated in-patient and out-patient groups however, the most common reasons for rarely appropriate studies in these investigations usually involve asymptomatic, low risk patients or those with known stable coronary disease [11, 13, 17]. As hospitalised patients tend to be symptomatic and have a greater number of risk factors than the out-patient population, it is unlikely that many in-patients will be in a low risk category. The rate of rarely appropriate diagnostic angiograms seen here was also lower here than that reported elsewhere. Studies by Hannan and Mohareb [12, 18] (who also performed their study in Ontario) both found higher rates of rarely appropriate use - 24.9 and 10.8 % respectively; however both of these studies excluded both investigation of acute coronary syndrome and valvular heart disease, which made of 72 % of our studies and are deemed appropriate by the AUC in all circumstances. A large study by Chan and colleagues assessed appropriateness of percutaneous coronary interventions and found an appropriateness rate of 98.6 % in patients having PCI for an acute indication, which is consistent with the low rarely appropriate rate in our study [19].

To our knowledge, this is the first study to investigate appropriateness of cardiac imaging across a range of modalities, and so provide a holistic picture of cardiac imaging appropriateness at individual centers. In our study, we found preferential usage of different imaging modalities between sites, with site 1 performing relatively few catheterizations compared to site 2, and site 3 performing more SPECT and less TEE compared to the other sites. The reasons for this could include site specific specialization, e.g. one site is the cardiac surgery center for all three sites and two of the three sites have specialized heart failure clinics, variable availability of cardiac imaging investigations, staff preference, or site specific factors which could not be accounted for in this study. With these variables in mind, studies that examine only one modality per site may miss important ordering patterns across other areas of cardiac imaging. For example, a study may note a high level of rarely appropriate use of stress echo, but miss the fact that, due to higher staff familiarity, they order many more SPECT with a high level of appropriateness. Recognising the range of imaging techniques in use, the ACCF has most recently published disease specific imaging AUC [20, 21] rather than modality specific guidelines to aid clinicians in choosing between the options available to them. This approach is useful for patients with chronic diseases, such as heart failure or chronic ischemic coronary disease, but would not capture patients having imaging without those diseases. With this in mind we believe that assessing and reporting on combined cardiac imaging appropriateness across modalities could serve as a useful tool for clinical departments to determine comprehensive imaging ordering patterns and appropriateness. Moreover, a combined appropriateness rate for all cardiac imaging modalities could become an important quality improvement indicator that takes into account site specific practicalities and preferences. Although low levels of rarely appropriate use were noted across the board in this study on acute care inpatients (6 and 3 % respectively for GIM and Cardiology), this may not be the case in other clinical environments, particularly in the ambulatory care environment. A quality improvement indicator could then be used to identify areas of improvement and to help evaluate interventions designed to improve appropriate cardiac imaging ordering.

Our study has important limitations that warrant mentioning. As our data was collected retrospectively from the written record, it is possible that the clinical indication for a test was not fully captured. However, we believe that we were able to draw on sufficient information from each case (admission note, consults, electronic requests and daily chart entries) to minimise this risk. Also, unlike non-invasive cardiac imaging, diagnostic catheterization requested by the GIM service would usually require an inpatient Cardiology consult before it could take place. Although this obviously affects the diagnostic catheterizations we have reported as being performed under the GIM team, we felt that this group of patients represented a different sample from those directly under the Cardiology service and that it was important to analyse them as such. In addition, as the GIM team would remain the most responsible physician and it is unusual for the Cardiology service to refuse an angiogram that has been specifically requested. Finally, this study was conducted at academic health sciences centers, whose results may differ from community practices.

## Conclusions

In conclusion, the ACCF AUC are able to capture the vast majority of clinical scenarios for multiple cardiac imaging modalities in this multi-centered retrospective study on general cardiology and GIM inpatients. Overall, the appropriateness ordering rate is high across all imaging modalities. We recommend that further work aimed at improving appropriate utilization of cardiac imaging resources should focus on investigations performed in the out-patient setting.

## Abbreviations

TTE: Transthoracic echo
TEE: Transesophageal echo
SPECT: Single-photon emission tomography myocardial perfusion imaging
ACCF: American College of Cardiology Foundation
AUC: Appropriate use criteria
GIM: General internal medicine
EMR: Electronic medical record
HTN: Hypertension
ACS: Acute coronary syndrome
CABG: Coronary artery by-pass graft
STEMI: ST-elevation myocardial infraction
NSTEMI: Non-ST elevation myocardial infarction
